# Supplementary material for: Using heterogeneity in the population structure of U.S. swine farms to compare transmission models for porcine epidemic diarrhoea
Source: Sci Rep. 2016 Mar 7;6:22248. doi: 10.1038/srep22248 (PMC4780089; doi:10.1038/srep22248)
Supplement: Supplementary Information [file srep22248-s1.pdf]

Supplementary Information: Using heterogeneity in the population structure of U.S. swine farms to compare transmission models for porcine epidemic diarrhoea

Eamon O'Dea Harry Snelson Shweta Bansal

February 17, 2016

Contents

Supplementary Figures 1

Supplementary Tables 10

Supplementary Note 17

1. Descriptive statistics of data sets . . . 17
2. Detailed data descriptions . . . 24
3. Simulations, correlation analysis, and sensitivity analysis . . . 26
4. Age-specific reporting bias . . . 27
5. Regularised regression and stability selection . . . 28
6. Time series regression modelling . . . 29
7. Software . . . 30

Supplementary Figures

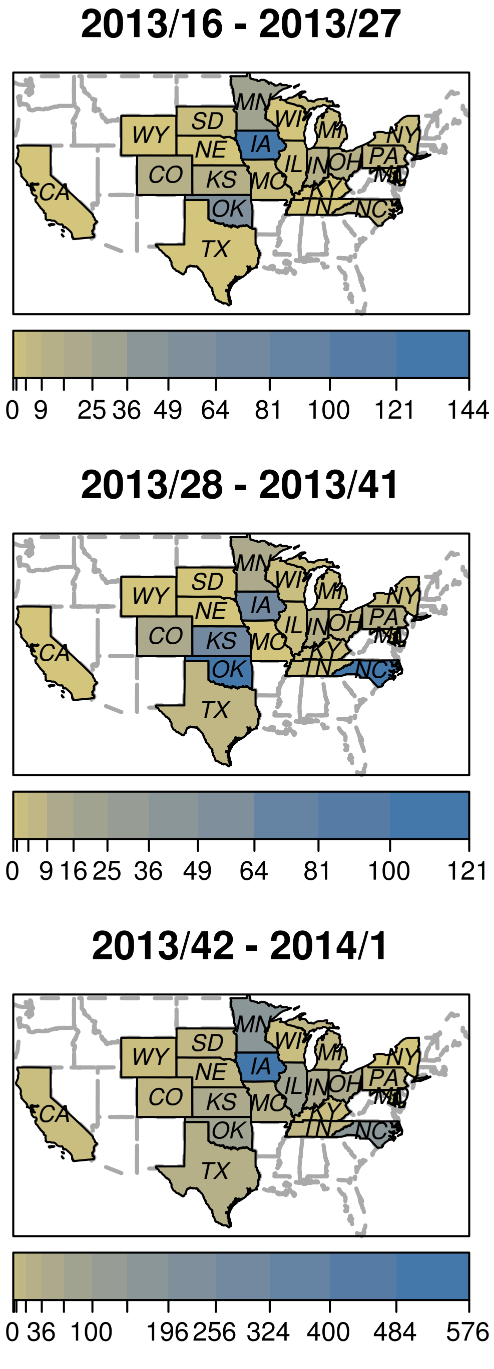

Figure S1: PEDV-positive accessions by state for three periods in 2013. Above each panel is the range of ISO weeks over which counts of positive accessions are aggregated to determine the fill color. The periods correspond roughly to spring, summer and autumn of 2013. Note that the scale of the colorbar is different for each panel and a lull occurred in the summer period for most states. States with no positive accessions in 2013 have dashed borders. This figure was created with the R package surveillance [1].

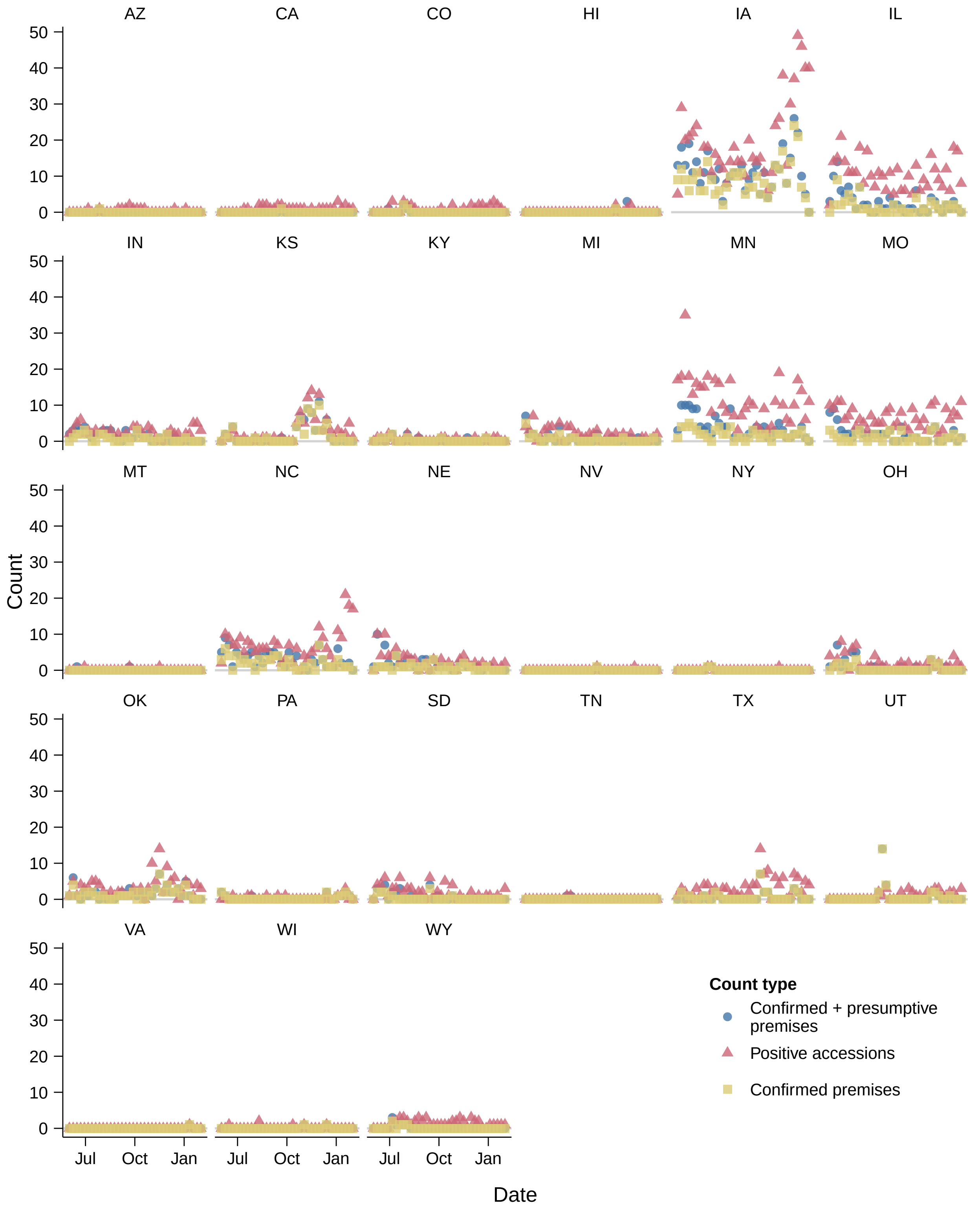

Figure S2: Counts of positive accessions and confirmed or presumptive positive premises by state in year 2014–2015. A confirmed positive premises is a premises where swine tested positive and have clinical signs. A presumptive positive premises is a premise where swine tested positive but have non-specific, unknown, or no clinical signs consistent with PED. The counts of positive accessions are similar to those of premises that are confirmed or presumptive.

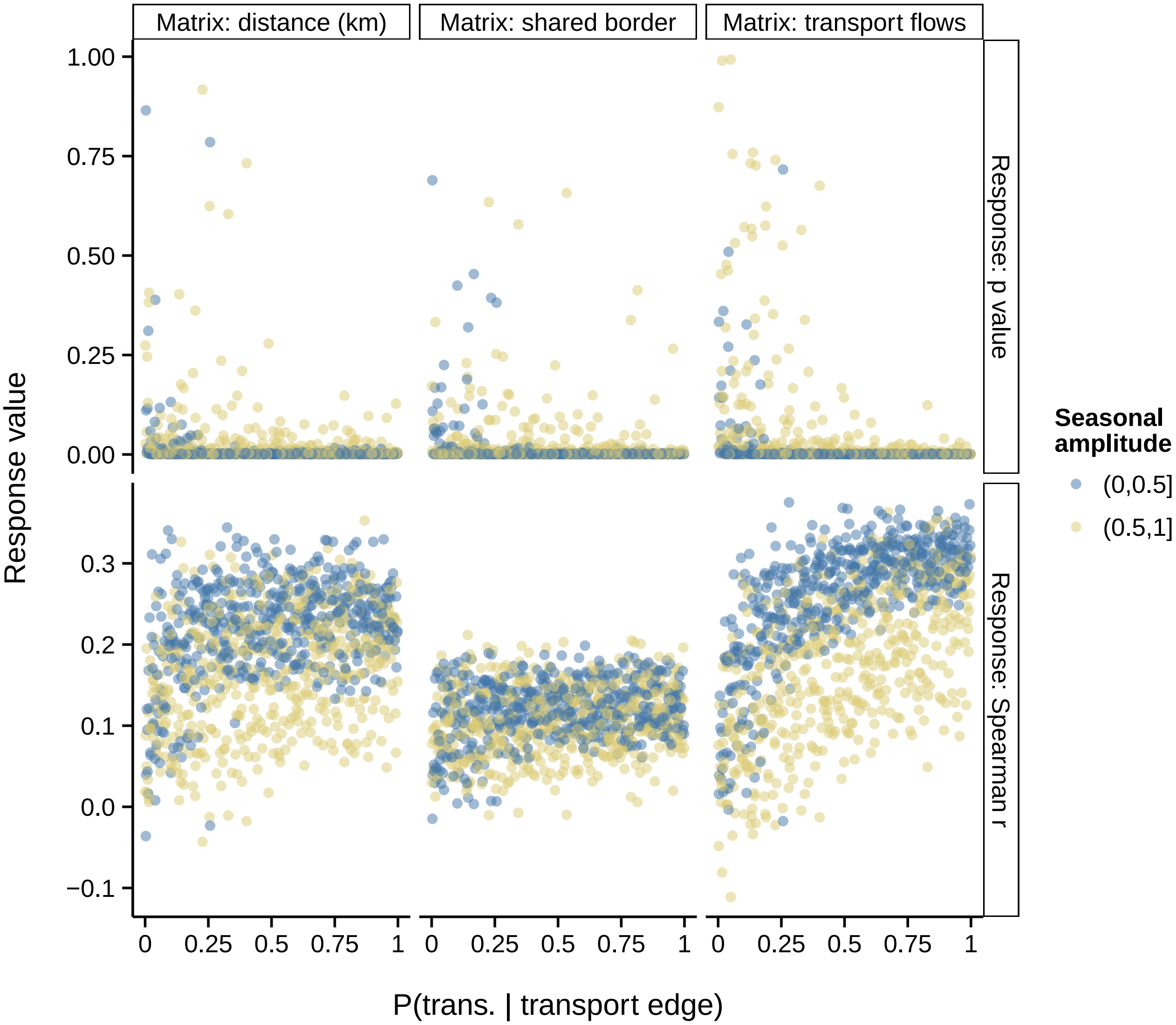

Figure S3: Matrix correlations and p values as a function of the transmission probability across transport edges in the contact network. For the distance and transport matrices, correlations increase with the transmission probability. In all cases, p values tend to decrease with increases in the transmission probability. These trends are clearer when the seasonal amplitude is below 0.5.

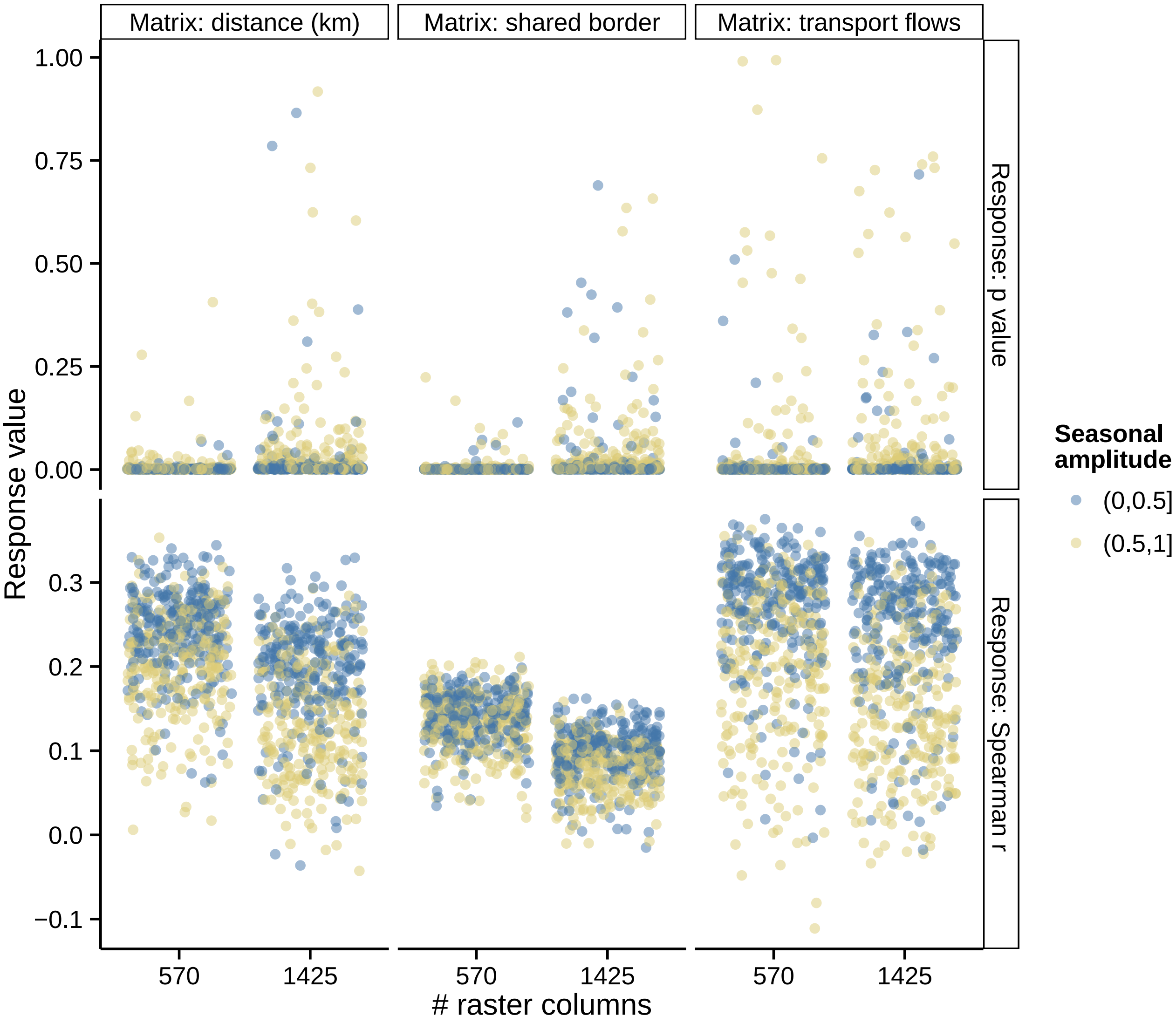

Figure S4: Matrix correlations and p values as a function of the number of the spatial resolution of the simulation. As the spatial resolution increases, the distances over which spatial edges occur in the contact network decreases. Matrix correlations were higher and p values were lower when spatial neighborhoods were larger. Seasonal amplitude has no noticeable effect.

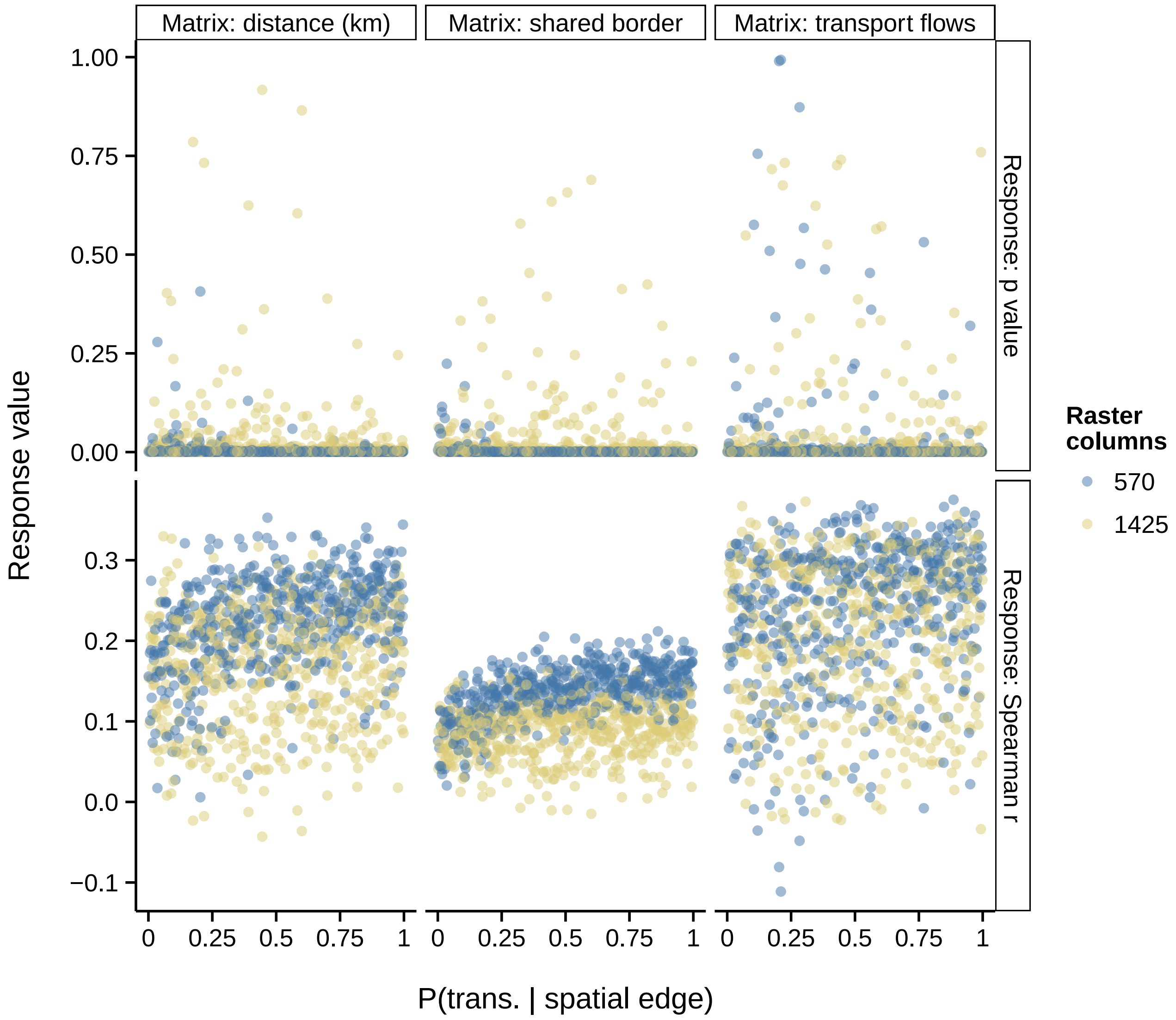

Figure S5: Matrix correlations and p values as a function of the probability of transmission across spatial edges in the contact network. On average, correlations are increasing and p values are decreasing. These trends are weak for the transport matrix.

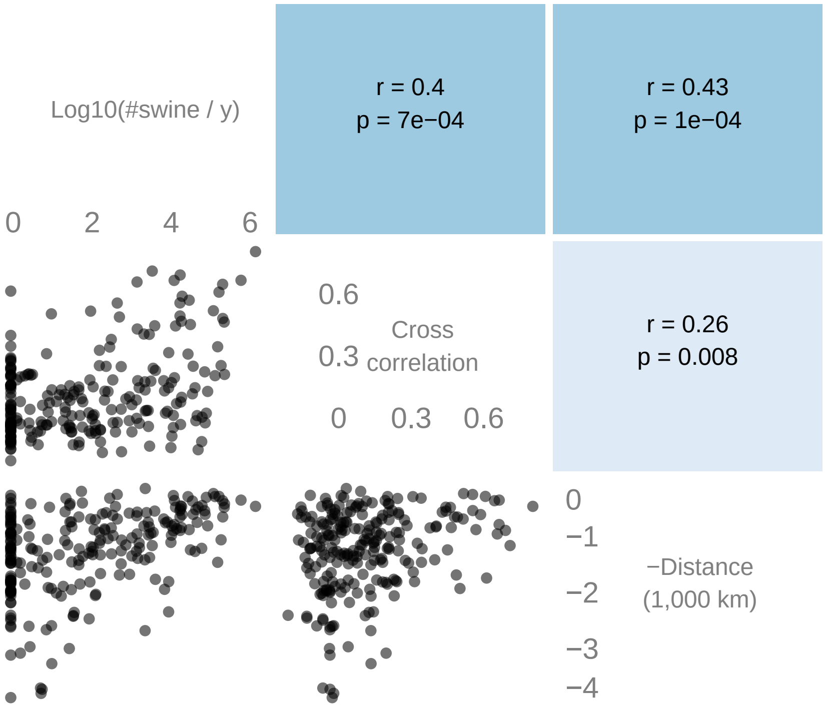

Figure S6: Scatter plots and Pearson correlations between pair-averaged (i.e., undirected) transport flows, cross correlations between time series of positive accessions, and negative geographic distances. The p values are from a Mantel tests.

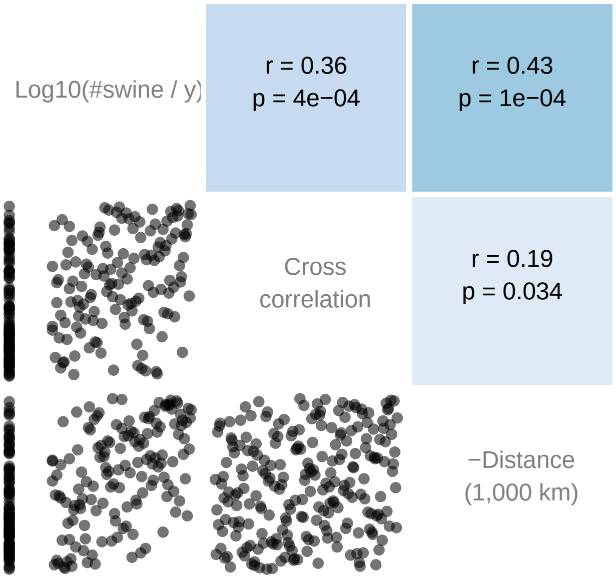

Figure S7: Rank scatter plots and Spearman correlations between transport flows, cross correlations between time series of positive accessions, and negative geographic distances. The p values are from a Mantel test.

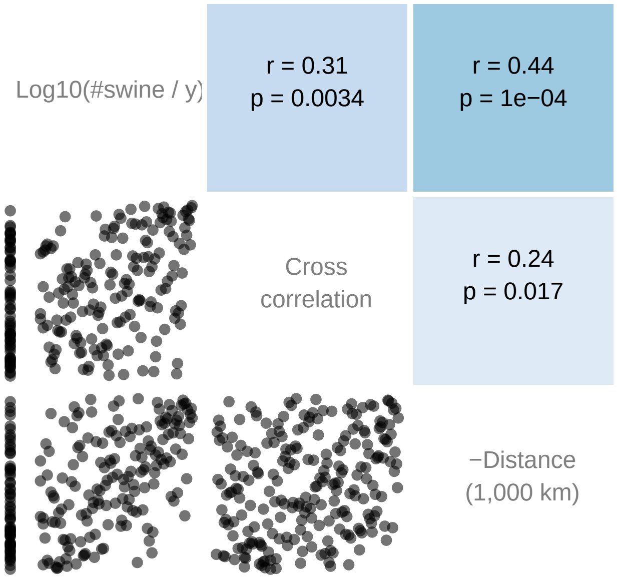

Figure S8: Rank scatter plots and Spearman correlations between pair-averaged (i.e., undirected) transport flows, cross correlations between time series of positive accessions, and negative geographic distances. The p values are from a Mantel test.

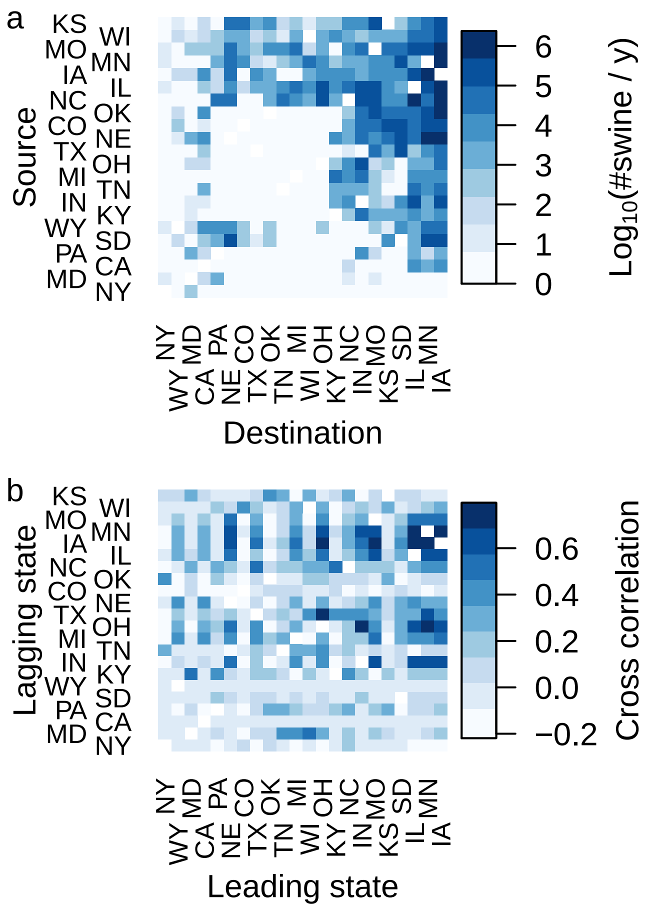

Figure S9: Comparison of swine transport flows and coupling of outbreak dynamics. (a) Annual swine transport flows from source states to destination states. (b) Cross correlations of PEDV positive accessions per week. Cross correlations are calculated as the correlations between positive accessions in the leading state with those in the lagging state in the previous week. Within-state values of flows and cross correlations are not included in the analysis and appear as white squares. In both panels, rows and columns are arranged to cluster together states with similar shipment flows.

Supplementary Tables

Table S1: Farm types and the age classes of swine typically present on them. Ones (zeros) indicate the presence (absence) of an age class on a particular farm type.

Farms type	Suckling	Nursery	Grower/Feeder	Sow/Boar
Farrow to wean	1	0	0	1
Farrow to finish	1	1	1	1
Finish only	0	0	1	0
Farrow to feeder	1	1	0	1
Nursery	0	1	0	0

Table S2: USDA ERS estimates of number of swine moved between states in 2001 for feeding and breeding. Origin in row, destination in column. Table 1 of 6.

	AL	AK	AZ	AR	CA	CO	CT	DE	DC
AL				247	5				
AK									
AZ					31				
AR	291		67		6				
CA									
CO					20				
CT									
DE									
DC									
FL				1	1				
GA	147332		1						
HI					1				
ID		2			51				
IL	92		28	62	496	120			
IN	46			12	8				
IA	170			999	14237				
KS	12			691	71	18144			
KY	130			18253					
LA					1				
ME					45				
MD					31			53	
MA									
MI					2				
MN				1		14917			
MS					350				
MO	2		12	48494	645	2134			
MT					815				
NE				185	8496				
NV					83				
NH	2								
NJ									
NM					5				
NY									
NC	27275							6600	
ND					190				
OH	7			21	108				
OK	1082			52520	16762				
OR					195				
PA					29				
RI									
SC									
SD	2355				404	229			
TN					805				
TX					183				
UT					4883				
VT									
VA									
WA		4			12				
WV									
WI					57	2379		5	
WY					11001	674			
Mexico									
Canada					6590				
Other states									

Table S3: USDA ERS estimates of number of swine moved between states in 2001 for feeding and breeding. Origin in row, destination in column. Table 2 of 6.

	FL	GA	HI	ID	IL	IN	IA	KS	KY
AL	413	22672			1200	980	244823		7270
AK									
AZ							1		
AR	11	16			13233	2430	241204	452	
CA					9415		4843		
CO					64790	28600	316678	248742	
CT		2							
DE									
DC									
FL		5124			39				
GA	2152				2805	5500	98494		16253
HI									
ID									
IL	234	86				113245	1424813	4278	16964
IN	4	877			101857		225425	69	2585
IA	69	431			190481	1435		9821	9694
KS		75			13947	14847	157764		258
KY		899			16205	45788	17658	1999	
LA	33								
ME		233			36				
MD					1				
MA									
MI					6745	81932	9232	6	35511
MN		550		2	1101	1380	1656184	5243	614
MS		1			461				
MO		177			223313	45820	2389932	21894	
MT							9900		
NE					34093	25375	1152749	85792	11762
NV									
NH		15			6				
NJ									
NM									
NY		2							
NC	87	13301			776744	408824	1216195	4003	3231
ND	1				8030		15397		969
OH		10			2601	193579	25456	157	549
OK		2656			47536	28475	1477925	82357	
OR					3		4		
PA					1283	15810	1016		
RI									
SC	17	106			60	1100			
SD				1590	2735		363885	4989	
TN	1699	2			34149	837	47036		3231
TX		205			317		82434	2872	
UT					2650		16005	960	
VT									
VA		36			1930	3320			
WA									
WV									
WI	20	31			18927	500	110098	1141	13765
WY				370	2396		84761	20	
Mexico									
Canada	800	74			24440	22028	1662037	3072	12343
Other states									

Table S4: USDA ERS estimates of number of swine moved between states in 2001 for feeding and breeding. Origin in row, destination in column. Table 3 of 6.

	LA	ME	MD	MA	MI	MN	MS	MO	MT
AL					1905	1025	13700	8	
AK									
AZ						8100			
AR	19				323	36907	1	589534	
CA						1413		1	
CO						203033	100	41934	
CT			1	79					
DE			1284						
DC									
FL	1		2						
GA					1	2458	3201	2400	
HI									
ID									
IL	500			3	22640	117647	37	98050	
IN			8			1347	55	306	
IA			37			860950	39	13890	
KS	4		4		354	22554	521	144338	
KY			6			2816	48	2406	
LA			208						
ME				986		27		44	
MD						2		12	12
MA			6						
MI						7759		178	
MN					976			6157	443
MS			1	1					
MO	5		290	1	22921	117548	139		
MT						4105			
NE			1015	1		725790	35	14074	
NV									
NH		275	60	141		1			
NJ						6			
NM									
NY			200	4					
NC					7207	55866	73220	231883	
ND						126132			
OH			27	8		2738	75	71	
OK						129194	35	397676	
OR			6			4			
PA			2120	24		117		34	
RI				11					
SC			7						
SD						306309		1	1172
TN						5917		601	
TX				7		5400		36867	
UT						3		2468	
VT									
VA			25			2			
WA									
WV			45	21					
WI			11		880	33922		1528	
WY			105			67822		254	23
Mexico									
Canada				3599		912246		2320	
Other states									

Table S5: USDA ERS estimates of number of swine moved between states in 2001 for feeding and breeding. Origin in row, destination in column. Table 4 of 6.

	NE	NV	NH	NJ	NM	NY	NC	ND	OH
AL	585						10000		851
AK									
AZ									
AR	950						10272		17
CA		9					35		
CO							1267		
CT									
DE				280					
DC									
FL				13					
GA	1200						23722		3313
HI									
ID		2							
IL	8725	2			35	9	35793		96974
IN		2					14629		
IA	34405						15448		10114
KS	59895				25		3786		177
KY							590		
LA							2382		
ME			57	13			18		
MD						17	9		2
MA			76			46			
MI							13220		
MN	46374					22	1248	1132	3866
MS	11					1	2920		
MO	18003	3113		1	8	10	6087		1361
MT									
NE							2075		
NV									
NH									
NJ									
NM									
NY				87			1		
NC	20900								130164
ND									
OH		1		17			8768		
OK							392		
OR							20		
PA				661					
RI									
SC							90834		
SD	98676							261	
TN							1320		
TX				8			25		
UT		32							
VT									
VA							34723		
WA									
WV					1				
WI	1081	2			38		830		859
WY	6484				10	7			320
Mexico									
Canada								16515	
Other states								2354	

Table S6: USDA ERS estimates of number of swine moved between states in 2001 for feeding and breeding. Origin in row, destination in column. Table 5 of 6.

	OK	OR	PA	RI	SC	SD	TN	TX	UT
AL						940	17645		
AK									
AZ						850			
AR	5596					1900	1275	35	
CA		144							
CO		1				208887			
CT									
DE									
DC									
FL					27		2359	2965	
GA	1387		7120		69		8235	450	
HI									
ID		213							
IL	2109	33	93	1	14	3295	7927	957	
IN		18				12330			
IA	1219	104	98			11698		10114	
KS	7676					359	66	2316	17
KY						1071			
LA									
ME									
MD			2253	3					
MA			2						
MI									
MN	18	2	2571			134537	173	45	
MS							129	8	
MO	5069	13	215			22608	9223	319	
MT						4837			
NE		2				141312			
NV									
NH									
NJ									
NM									
NY									
NC	2015		54309		176474	15800	29879	3	
ND						1839			
OH						1			
OK		9				22141			
OR									
PA									
RI									
SC									
SD	309	3	2733					25	
TN									
TX		1				175095			
UT		1				9			
VT									
VA									
WA		101							
WV			70						
WI	671	1	480		62	1008	7	59	
WY	250		7810			7501			11
Mexico									
Canada						145137			
Other states									

Table S7: USDA ERS estimates of number of swine moved between states in 2001 for feeding and breeding. Origin in row, destination in column. Table 6 of 6.

	VT	VA	WA	WV	WI	WY	Mexico	Canada	Other states
AL									
AK									
AZ									
AR									
CA									
CO				1		234			
CT									
DE									
DC									
FL		8							
GA		450			762				
HI									
ID									
IL		90			16207				
IN				12					
IA					1219	27			21896
KS				19	21	24			34300
KY									
LA						7			
ME									
MD		341		22					
MA	2	4							
MI				3					
MN			1	14	30893				1
MS									153
MO			2		127				
MT						47			
NE						17			
NV									
NH									
NJ									
NM									
NY									
NC		136736			2425				
ND						10			
OH				26					
OK				1		38			
OR									
PA				16					
RI									
SC									
SD						101		2181	
TN									
TX						2			
UT									
VT									
VA				3					
WA									
WV									
WI		61				62			
WY									
Mexico									
Canada									
Other states									

Supplementary Note

1. Descriptive statistics of data sets

The tables below provide the number of observations n, the number of missing values, the number of unique values, and the mean. Depending on their distributions, variables are further described with some subset of quantiles, order statistics, histograms, and probability mass functions.

Variables in Mantel tests
4 Variables 462 Observations

Transport flow, log10(#swine / y + 1)

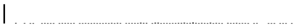

n	missing	unique	Mean	.05	.10	.25	.50	.75	.90	.95
462	0	246	1.93	0.000	0.000	0.000	1.447	3.788	4.729	5.196

```
lowest : 0.0000 0.3010 0.4771 0.6021 0.6990
highest: 6.0850 6.1538 6.1697 6.2191 6.3784
```

Cross correlation in positive accessions

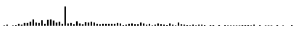

```
       n  missing   unique       Mean      .05      .10      .25      .50
     462        0      413     0.1158 -0.13346 -0.11225 -0.05071  0.04438
     .75      .90      .95
 0.25525  0.42874  0.57676
lowest : -0.2190 -0.2078 -0.1994 -0.1994 -0.1791
highest:  0.7353  0.7410  0.7614  0.7898  0.7959
```

-Geographic distance (km)

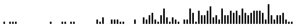

n	missing	unique	Mean	.05	.10	.25	.50	.75	.90	.95
462	0	231	-1271	-2609.7	-2186.9	-1716.7	-1133.6	-724.8	-486.0	-366.1

```
lowest : -3843.0 -3769.1 -3698.4 -3676.4 -3250.2
highest:  -312.5  -297.6  -281.3  -242.9  -193.3
```

Shared border

n	missing	unique	Sum	Mean
462	0	2	76	0.1645

Variables used in stability selection with responses of any positive accessions
26 Variables 42 Observations

Any positive accessions

n	missing	unique
42	0	2

```
FALSE (20, 48%), TRUE (22, 52%)
```

Log(#farms)

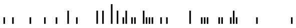

n	missing	unique	Mean	.05	.10	.25	.50	.75	.90	.95
42	0	40	5.474	2.962	3.556	4.580	5.277	6.757	7.548	7.609

```
lowest : 2.303 2.565 2.944 3.296 3.532
highest: 7.554 7.598 7.610 8.100 8.929
```

Log(mean over counties of #farms / km^2)

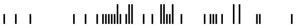

n	missing	unique	Mean	.05	.10	.25	.50	.75	.90	.95
42	0	42	-6.292	-9.164	-7.885	-7.248	-6.234	-4.987	-4.298	-3.873

```
lowest : -9.906 -9.572 -9.214 -8.218 -7.907
highest: -4.288 -4.282 -3.852 -3.774 -2.982
```

Log(mean over counties with farms of #farms / km^2)

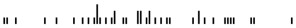

n	missing	unique	Mean	.05	.10	.25	.50	.75	.90	.95
42	0	42	-5.915	-8.321	-7.534	-6.764	-5.954	-4.839	-4.203	-3.833

```
lowest : -8.586 -8.489 -8.349 -7.790 -7.571
highest: -4.199 -4.105 -3.819 -3.715 -2.982
```

Log(median over counties of #farms / km^2)

n	missing	unique	Mean	.05	.10	.25	.50	.75	.90	.95
42	0	40	-6.974	-10.795	-8.626	-8.016	-6.993	-5.699	-4.850	-4.369

```
lowest : -10.850  -9.752  -8.631  -8.588  -8.574
highest:  -4.814  -4.416  -4.366  -4.219  -3.115
```

Log(maximum over counties of #farms / km^2)

n	missing	unique	Mean	.05	.10	.25	.50	.75	.90	.95
42	0	42	-4.365	-7.400	-6.210	-5.301	-4.582	-3.299	-2.262	-1.834

```
lowest : -7.732 -7.506 -7.455 -6.364 -6.264
highest: -2.262 -2.058 -1.822 -1.563 -1.456
```

Log(swine inventory in year 2012)

n	missing	unique	Mean	.05	.10	.25	.50	.75	.90	.95
42	0	42	5.327	1.496	1.807	3.790	5.301	7.077	8.219	8.917

```
lowest : 0.2624 0.9933 1.4951 1.5041 1.7918
highest: 8.2428 8.4338 8.9425 9.1050 9.9330
```

Log(pig crop)

n	missing	unique	Mean	.05	.10	.25	.50	.75	.90	.95
42	0	42	6.007	1.731	2.281	4.274	6.573	8.038	8.953	9.408

```
lowest : 0.3365 0.5306 1.7192 1.9459 2.2721
highest: 8.9564 9.2229 9.4176 9.8014 9.9241
```

Log(inshipments)

n	missing	unique	Mean	.05	.10	.25	.50	.75	.90	.95
42	0	37	4.053	0.004766	0.182322	2.165058	4.429042	5.875295	6.714020	7.895151

```
lowest :  0.00000  0.09531  0.18232  0.69315  1.38629
highest:  6.71659  7.83320  7.89841  8.98457 10.08581
```

Log(marketings)

n	missing	unique	Mean	.05	.10	.25	.50	.75	.90	.95
42	0	42	6.224	1.866	2.184	4.802	6.612	8.174	9.001	9.699

```
lowest :  0.5878  1.8310  1.8563  2.0541  2.1518
highest:  9.0014  9.4175  9.7133  9.8706 10.6246
```

Weighted mean over counties of whether a county is in resource region 1

n	missing	unique	Mean
42	0	8	0.1672

```
0 (33, 79%), 0.334405144694534 (1, 2%), 0.661064425770308 (1, 2%)
0.667582417582418 (1, 2%), 0.705559368565546 (1, 2%)
0.796841785605831 (1, 2%), 0.857545839210155 (1, 2%), 1 (3, 7%)
```

Weighted mean over counties of whether a county is in resource region 2

n	missing	unique	Mean
42	0	6	0.1609

```
0 (33, 79%), 0.190403887033101 (1, 2%), 0.198352779684283 (1, 2%)
0.481981981981982 (1, 2%), 0.888992537313433 (1, 2%), 1 (5, 12%)
```

Weighted mean over counties of whether a county is in resource region 3

n	missing	unique	Mean
42	0	8	0.06456

```
0 (35, 83%), 0.0127543273610689 (1, 2%), 0.0453781512605042 (1, 2%)
0.332417582417582 (1, 2%), 0.37888198757764 (1, 2%)
0.425925925925926 (1, 2%), 0.516129032258065 (1, 2%), 1 (1, 2%)
```

Weighted mean over counties of whether a county is in resource region 4

n	missing	unique	Mean
42	0	7	0.08144

```
0 (36, 86%), 0.293557422969188 (1, 2%), 0.397515527950311 (1, 2%)
0.4 (1, 2%), 0.662087912087912 (1, 2%), 0.667447306791569 (1, 2%)
1 (1, 2%)
```

Weighted mean over counties of whether a county is in resource region 5

n	missing	unique	Mean	.05	.10	.25	.50	.75	.90	.95
42	0	14	0.1038	0.00000	0.00000	0.00000	0.00000	0.03363	0.46252	0.65861

```
0 (29, 69%), 0.00576217915138816 (1, 2%), 0.018018018018018 (1, 2%)
0.0388349514563107 (1, 2%), 0.0960878517501716 (1, 2%)
0.111007462686567 (1, 2%), 0.115537848605578 (1, 2%)
0.142454160789845 (1, 2%), 0.337912087912088 (1, 2%)
0.476363636363636 (1, 2%), 0.525925925925926 (1, 2%)
0.665594855305466 (1, 2%), 0.825726141078838 (1, 2%), 1 (1, 2%)
```

Weighted mean over counties of whether a county is in resource region 6

n	missing	unique	Mean	.05	.10	.25	.50	.75	.90	.95
42	0	11	0.123	0.0000	0.0000	0.0000	0.0000	0.0000	0.4974	0.8668

```
0 (32, 76%), 0.016597510373444 (1, 2%), 0.175257731958763 (1, 2%)
0.192037470725995 (1, 2%), 0.206611570247934 (1, 2%)
0.474074074074074 (1, 2%), 0.5 (1, 2%), 0.854368932038835 (1, 2%)
0.867469879518072 (1, 2%), 0.884462151394422 (1, 2%)
0.994237820848612 (1, 2%)
```

Weighted mean over counties of whether a county is in resource region 7

n	missing	unique	Mean	.05	.10	.25	.50	.75	.90	.95
42	0	10	0.1353	0.0000	0.0000	0.0000	0.0000	0.0000	0.8352	0.8493

```
0 (33, 79%), 0.106796116504854 (1, 2%), 0.132530120481928 (1, 2%)
0.140515222482436 (1, 2%), 0.806451612903226 (1, 2%)
0.838383838383838 (1, 2%), 0.845238095238095 (1, 2%)
0.849462365591398 (1, 2%), 0.962962962962963 (1, 2%), 1 (1, 2%)
```

Weighted mean over counties of whether a county is in resource region 8

n	missing	unique	Mean	.05	.10	.25	.50	.75	.90	.95
42	0	11	0.109	0.00000	0.00000	0.00000	0.00000	0.02778	0.45784	0.59870

```
0 (31, 74%), 0.037037037037037 (1, 2%), 0.150537634408602 (1, 2%)
0.154761904761905 (1, 2%), 0.161616161616162 (1, 2%)
0.193548387096774 (1, 2%), 0.22360248447205 (1, 2%)
0.483870967741935 (1, 2%), 0.574074074074074 (1, 2%), 0.6 (1, 2%)
1 (2, 5%)
```

Weighted mean over counties of whether a county is in resource region 9

n	missing	unique	Mean
42	0	5	0.05475

```
0 (38, 90%), 0.157676348547718 (1, 2%), 0.523636363636364 (1, 2%)
0.793388429752066 (1, 2%), 0.824742268041237 (1, 2%)
```

Log (positive accessions), weighted by shared border

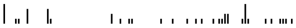

n	missing	unique	Mean	.05	.10	.25	.50	.75	.90	.95
42	0	34	3.591	-1.0000	-0.5756	0.3219	4.6585	6.2385	7.0276	7.3729

```
lowest : -1.0000 -0.5850 -0.4912 -0.2630  0.3219
highest:  7.0470  7.3038  7.3765  7.5107  7.6884
```

Log (positive accessions), weighted by directed flows

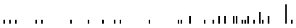

n	missing	unique	Mean	.05	.10	.25	.50	.75	.90	.95
42	0	42	21.3	3.746	6.644	17.326	24.388	27.330	29.821	29.971

```
lowest :  2.459  3.047  3.644  5.687  6.384
highest: 29.823 29.924 29.974 30.071 30.639
```

Log (positive accessions), weighted by directed flows$^{0.5}$

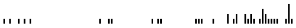

n	missing	unique	Mean	.05	.10	.25	.50	.75	.90	.95
42	0	42	14.74	2.594	3.714	12.226	17.094	18.529	19.861	19.913

```
lowest :  1.452  1.987  2.576  2.934  3.265
highest: 19.867 19.909 19.913 19.943 20.238
```

Log (positive accessions), weighted by directed flows$^{0.25}$

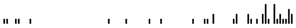

n	missing	unique	Mean	.05	.10	.25	.50	.75	.90	.95
42	0	42	11.61	1.673	2.805	10.990	13.652	14.370	15.025	15.087

```
lowest :  0.9966  1.2395  1.6655  1.8149  2.4237
highest: 15.0411 15.0667 15.0884 15.1906 15.3049
```

Log (positive accessions), weighted by directed flows$^{0.125}$

n	missing	unique	Mean	.05	.10	.25	.50	.75	.90	.95
42	0	42	10.13	1.097	2.674	9.857	11.869	12.503	12.716	12.774

```
lowest :  0.7848  0.8986  1.0941  1.1618  2.3620
highest: 12.7186 12.7648 12.7742 12.9473 13.0080
```

Log (positive accessions), weighted by directed flows$^{0.0625}$

n	missing	unique	Mean	.05	.10	.25	.50	.75	.90	.95
42	0	42	9.428	0.8323	2.6110	9.4022	11.0280	11.5403	11.6722	11.7493

```
lowest :  0.6833  0.7381  0.8307  0.8624  2.3341
highest: 11.6724 11.7110 11.7513 11.8737 11.9198
```

Data for stability selection with response of total positive accessions
25 Variables 22 Observations

Log(total positive accessions)

n	missing	unique	Mean	.05	.10	.25	.50	.75	.90	.95
22	0	19	2.857	0.00000	0.06931	1.44208	3.05462	4.21639	5.46536	5.71144

```
-1.09861228866811 (1, 5%), 0 (2, 9%), 0.693147180559945 (1, 5%)
1.38629436111989 (2, 9%), 1.6094379124341 (2, 9%)
1.79175946922805 (1, 5%), 2.19722457733622 (1, 5%)
2.89037175789616 (1, 5%), 3.2188758248682 (1, 5%)
3.3322045101752 (1, 5%), 3.40119738166216 (1, 5%)
4.06044301054642 (1, 5%), 4.07753744390572 (1, 5%)
4.26267987704132 (1, 5%), 4.97673374242057 (1, 5%)
5.32787616878958 (1, 5%), 5.48063892334199 (1, 5%)
5.72358510195238 (1, 5%), 6.5206211275587 (1, 5%)
```

Log(#farms)

n	missing	unique	Mean	.05	.10	.25	.50	.75	.90	.95
22	0	22	6.481	4.044	5.096	5.648	6.701	7.436	7.609	8.075

```
lowest : 3.769 3.989 5.081 5.226 5.557
highest: 7.554 7.598 7.610 8.100 8.929
```

Log(mean over counties of #farms / km^2)

n	missing	unique	Mean	.05	.10	.25	.50	.75	.90	.95
22	0	22	-5.376	-7.455	-7.384	-6.036	-5.020	-4.400	-3.895	-3.778

```
lowest : -8.218 -7.458 -7.404 -7.205 -6.106
highest: -4.288 -4.282 -3.852 -3.774 -2.982
```

Log(mean over counties with farms of #farms / km^2)

n	missing	unique	Mean	.05	.10	.25	.50	.75	.90	.95
22	0	22	-5.168	-6.948	-6.809	-5.797	-4.860	-4.259	-3.847	-3.720

```
lowest : -7.790 -6.955 -6.811 -6.792 -5.967
highest: -4.199 -4.105 -3.819 -3.715 -2.982
```

Log(median over counties of #farms / km^2)

n	missing	unique	Mean	.05	.10	.25	.50	.75	.90	.95
22	0	22	-5.891	-8.323	-7.905	-6.317	-5.729	-5.182	-4.371	-4.226

```
lowest : -8.588 -8.344 -7.927 -7.708 -6.695
highest: -4.814 -4.416 -4.366 -4.219 -3.115
```

Log(maximum over counties of #farms / km^2)

n	missing	unique	Mean	.05	.10	.25	.50	.75	.90	.95
22	0	22	-3.474	-5.609	-5.366	-4.489	-3.301	-2.269	-1.845	-1.576

```
lowest : -6.364 -5.622 -5.371 -5.321 -4.846
highest: -2.262 -2.058 -1.822 -1.563 -1.456
```

Log(swine inventory in year 2012)

n	missing	unique	Mean	.05	.10	.25	.50	.75	.90	.95
22	0	22	6.825	4.203	4.474	5.760	7.064	7.985	8.892	9.097

```
lowest : 2.367 4.190 4.454 4.654 5.011
highest: 8.243 8.434 8.942 9.105 9.933
```

Log(pig crop)

n	missing	unique	Mean	.05	.10	.25	.50	.75	.90	.95
22	0	22	7.566	4.190	4.884	6.655	7.996	8.921	9.398	9.782

```
lowest : 3.138 4.159 4.787 5.753 6.510
highest: 8.956 9.223 9.418 9.801 9.924
```

Log(inshipments)

n	missing	unique	Mean	.05	.10	.25	.50	.75	.90	.95
22	0	22	5.711	2.492	2.779	4.518	5.825	6.681	7.892	8.930

```
lowest :  1.386  2.485  2.637  4.060  4.369
highest:  6.717  7.833  7.898  8.985 10.086
```

Log(marketings)

n	missing	unique	Mean	.05	.10	.25	.50	.75	.90	.95
22	0	22	7.781	5.021	5.548	6.672	8.102	8.987	9.684	9.863

```
lowest :  3.638  4.995  5.509  5.892  6.586
highest:  9.001  9.418  9.713  9.871 10.625
```

Weighted mean over counties of whether a county is in resource region 1

n	missing	unique	Mean
22	0	8	0.3192

```
0 (13, 59%), 0.334405144694534 (1, 5%), 0.661064425770308 (1, 5%)
0.667582417582418 (1, 5%), 0.705559368565546 (1, 5%)
0.796841785605831 (1, 5%), 0.857545839210155 (1, 5%), 1 (3, 14%)
```

Weighted mean over counties of whether a county is in resource region 2

n	missing	unique	Mean
22	0	6	0.2164

```
0 (15, 68%), 0.190403887033101 (1, 5%), 0.198352779684283 (1, 5%)
0.481981981981982 (1, 5%), 0.888992537313433 (1, 5%), 1 (3, 14%)
```

Weighted mean over counties of whether a county is in resource region 3

n	missing	unique	Mean
22	0	6	0.05433

```
0 (17, 77%), 0.0127543273610689 (1, 5%), 0.0453781512605042 (1, 5%)
0.332417582417582 (1, 5%), 0.37888198757764 (1, 5%)
0.425925925925926 (1, 5%)
```

Weighted mean over counties of whether a county is in resource region 4

n	missing	unique	Mean
22	0	6	0.1373

```
0 (17, 77%), 0.293557422969188 (1, 5%), 0.397515527950311 (1, 5%)
0.662087912087912 (1, 5%), 0.667447306791569 (1, 5%), 1 (1, 5%)
```

Weighted mean over counties of whether a county is in resource region 5

n	missing	unique	Mean
22	0	9	0.08424

```
0 (14, 64%), 0.00576217915138816 (1, 5%), 0.018018018018018 (1, 5%)
0.0960878517501716 (1, 5%), 0.111007462686567 (1, 5%)
0.142454160789845 (1, 5%), 0.337912087912088 (1, 5%)
0.476363636363636 (1, 5%), 0.665594855305466 (1, 5%)
```

Weighted mean over counties of whether a county is in resource region 6

n	missing	unique	Mean
22	0	4	0.07665

```
0 (19, 86%), 0.192037470725995 (1, 5%), 0.5 (1, 5%)
0.994237820848612 (1, 5%)
```

Weighted mean over counties of whether a county is in resource region 7

n	missing	unique	Mean
22	0	3	0.045

```
0 (20, 91%), 0.140515222482436 (1, 5%), 0.849462365591398 (1, 5%)
```

Weighted mean over counties of whether a county is in resource region 8

n	missing	unique	Mean
22	0	4	0.0431

```
0 (19, 86%), 0.150537634408602 (1, 5%), 0.22360248447205 (1, 5%)
0.574074074074074 (1, 5%)
```

Weighted mean over counties of whether a county is in resource region 9

n	missing	unique	Mean
22	0	2	0.0238

```
0 (21, 95%), 0.523636363636364 (1, 5%)
```

Log (positive accessions), weighted by shared border

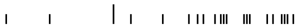

n	missing	unique	Mean	.05	.10	.25	.50	.75	.90	.95
22	0	22	4.962	0.417	2.229	3.898	5.531	6.719	7.369	7.504

```
lowest : -1.0000  0.3219  2.2224  2.2870  2.8074
highest:  7.0470  7.3038  7.3765  7.5107  7.6884
```

Log (positive accessions), weighted by directed flows

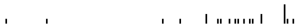

n	missing	unique	Mean	.05	.10	.25	.50	.75	.90	.95
22	0	22	25.39	12.58	20.85	24.27	26.58	29.40	29.97	30.07

```
lowest :  8.984 12.157 20.718 22.022 24.010
highest: 29.823 29.924 29.974 30.071 30.639
```

Log (positive accessions), weighted by directed flows$^{0.5}$

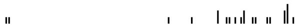

n	missing	unique	Mean	.05	.10	.25	.50	.75	.90	.95
22	0	22	17.46	8.835	15.139	17.202	18.190	19.646	19.913	19.942

```
lowest :  8.325  8.508 15.047 15.959 17.070
highest: 19.867 19.909 19.913 19.943 20.238
```

Log (positive accessions), weighted by directed flows$^{0.25}$

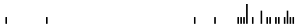

n	missing	unique	Mean	.05	.10	.25	.50	.75	.90	.95
22	0	22	13.69	8.487	12.519	13.787	14.225	14.852	15.086	15.185

```
lowest :  7.058  8.277 12.461 13.034 13.732
highest: 15.041 15.067 15.088 15.191 15.305
```

Log (positive accessions), weighted by directed flows$^{0.125}$

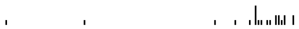

n	missing	unique	Mean	.05	.10	.25	.50	.75	.90	.95
22	0	22	11.91	8.416	11.263	12.110	12.307	12.641	12.773	12.939

```
lowest :  6.439  8.269 11.221 11.639 12.005
highest: 12.719 12.765 12.774 12.947 13.008
```

Log (positive accessions), weighted by directed flows$^{0.0625}$

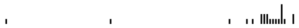

n	missing	unique	Mean	.05	.10	.25	.50	.75	.90	.95
22	0	22	11.05	8.384	10.649	11.253	11.390	11.641	11.707	11.866

```
lowest :  6.158  8.266 10.614 10.969 11.087
highest: 11.670 11.672 11.711 11.874 11.920
```

Distribution of positive accessions over states of origin by age class
4 Variables 20 Observations

Grower/Finisher

n	missing	unique	Mean	.05	.10	.25	.50	.75	.90	.95
20	0	14	25.1	0.00	0.00	0.75	8.00	16.50	47.20	76.20

	0	1	2	4	6	10	11	12	13	27	41	45	67	251
Frequency	5	2	1	1	1	1	2	1	1	1	1	1	1	1
%	25	10	5	5	5	5	10	5	5	5	5	5	5	5

Nursery

n	missing	unique	Mean	.05	.10	.25	.50	.75	.90	.95
20	0	15	19.95	0.00	0.00	0.75	6.50	15.50	52.20	75.85

	0	1	3	4	6	7	8	10	12	14	20	42	50	72	149
Frequency	5	2	1	1	1	1	1	1	1	1	1	1	1	1	1
%	25	10	5	5	5	5	5	5	5	5	5	5	5	5	5

Sow/Boar

n	missing	unique	Mean	.05	.10	.25	.50	.75	.90	.95
20	0	12	9.7	0.00	0.00	0.00	2.50	8.25	37.20	39.60

	0	1	2	3	4	5	8	9	30	37	39	51
Frequency	8	1	1	1	1	2	1	1	1	1	1	1
%	40	5	5	5	5	10	5	5	5	5	5	5

Suckling

n	missing	unique	Mean	.05	.10	.25	.50	.75	.90	.95
20	0	14	21.8	0.0	0.9	2.0	5.0	20.5	59.5	101.6

	0	1	2	3	4	6	10	11	20	22	52	55	100	132
Frequency	2	2	4	1	1	1	1	2	1	1	1	1	1	1
%	10	10	20	5	5	5	5	10	5	5	5	5	5	5

Variables in logistic regression of the proportion of positive accessions from the suckling age class

3 Variables 20 Observations

Observed proportion suckling

n	missing	unique	Mean	.05	.10	.25	.50	.75	.90	.95
20	0	15	0.4045	0.0000	0.1385	0.2266	0.2679	0.4306	1.0000	1.0000

```
0 (2, 10%), 0.153846153846154 (1, 5%), 0.16 (1, 5%)
0.212765957446809 (1, 5%), 0.231173380035026 (1, 5%)
0.244444444444444 (1, 5%), 0.25 (1, 5%), 0.263157894736842 (2, 10%)
0.272727272727273 (1, 5%), 0.373134328358209 (1, 5%)
0.379310344827586 (1, 5%), 0.37956204379562 (1, 5%)
0.407407407407407 (1, 5%), 0.5 (1, 5%), 1 (4, 20%)
```

Number of positive accessions with known age class

n	missing	unique	Mean	.05	.10	.25	.50	.75	.90	.95
20	0	18	76.55	1.00	1.00	3.75	23.50	64.00	214.90	283.15

	1	2	3	4	6	8	13	22	25	29	38	45	54	94	137	209	268	571
Frequency	3	1	1	1	1	1	1	1	1	1	1	1	1	1	1	1	1	1
%	15	5	5	5	5	5	5	5	5	5	5	5	5	5	5	5	5	5

Model sampling probability of suckling

n	missing	unique	Mean	.05	.10	.25	.50	.75	.90	.95
20	0	20	0.1313	0.06501	0.08031	0.09393	0.11338	0.13888	0.20801	0.27182

```
lowest : 0.05961 0.06530 0.08198 0.08972 0.09151
highest: 0.14607 0.19812 0.20097 0.27139 0.28010
```

Variables used in likelihood ratio test of within-state flows

7 Variables 1776 Observations

Positive accessions this week

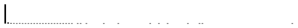

n	missing	unique	Mean	.05	.10	.25	.50	.75	.90	.95
1776	0	36	1.153	0	0	0	0	0	2	6

lowest : 0 1 2 3 4, highest: 54 61 65 66 96

Log(positive accessions last week + 0.5)

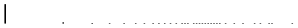

n	missing	unique	Mean	.05	.10	.25	.50	.75	.90	.95
1776	0	35	-0.3485	-0.6931	-0.6931	-0.6931	-0.6931	-0.6931	0.9163	1.8718

lowest : -0.6931 0.4055 0.9163 1.2528 1.5041
highest: 3.9416 4.1190 4.1821 4.1972 4.5695

Scaled log[(median farm density among counties having farms) (# farms)^2]

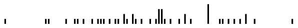

n	missing	unique	Mean	.05	.10	.25
1776	0	48	1.409e-17	-1.11739	-0.83959	-0.43886

.50	.75	.90	.95
0.03578	0.56114	0.90760	0.98326

lowest : -1.6059 -1.1671 -1.1174 -0.9422 -0.8396
highest: 0.9076 0.9698 0.9833 1.0571 1.4788

Scaled log[2 (#swine moved within state) / y]

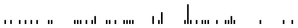

n	missing	unique	Mean	.05	.10	.25	.50	.75	.90	.95
1776	0	48	1.949e-16	-1.1805	-1.0482	-0.4992	0.1086	0.5008	0.8120	1.0686

lowest : -1.3168 -1.2602 -1.1805 -1.1172 -1.0482
highest: 0.8120 0.8454 1.0686 1.2861 1.3881

Centred week

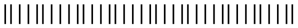

n	missing	unique	Mean	.05	.10	.25	.50	.75	.90	.95
1776	0	37	0.5	-16.5	-14.5	-8.5	0.5	9.5	15.5	17.5

lowest : -17.5 -16.5 -15.5 -14.5 -13.5
highest: 14.5 15.5 16.5 17.5 18.5

State

n	missing	unique
1776	0	48

lowest : AL AR AZ CA CO, highest: VT WA WI WV WY

Centred offset

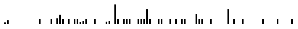

n	missing	unique	Mean	.05	.10	.25
1776	0	337	9.502e-17	-1.64789	-1.51607	-0.92311

.50	.75	.90	.95
-0.03373	0.92442	1.58652	2.10097

lowest : -2.502 -2.496 -2.486 -2.479 -2.472
highest: 1.587 1.745 2.101 2.371 2.643

---

2. Detailed data descriptions

Transport flows

Our data on shipment of live swine was an estimate of the total number of swine moved between all ordered pairs of states over the course of a year. We refer to these estimates as transport flows. They were generated by the USDA Economic Research Service [2] and are available on the web.[1] They are also printed in Supplementary Tables S2–S7.

State-to-state distances

R's[3] datasets package provided the coordinates of the approximate geographic centres of each state. We used these coordinates to calculate the distance between pairs of states via the haversine formula. This formula

[1] http://webarchives.cdlib.org/sw1rf5mh0k/http://ers.usda.gov/Data/InterstateLivestockMovements/StateShipments.xls

uses a spherical model of the Earth to account for the curvature of the Earth's surface on the shortest path along the surface between two locations.

Positive accessions

We obtained the case data from the January 8 version of a publicly available report of laboratory testing activity [4] via the "Number of New Cases Reported" link on the webpage of the American Association of Swine Veterinarians (AASV). We used the data in Tables 2 and 4 in this report. Table 2 contained the number of positive diagnostic case submissions stratified by the state of origin and the week of submission. Table 4 contained the number of positive diagnostic case submissions stratified by the state of origin and the age class of the swine from which the tissue sample was taken.

The precise meaning of the values in the time series changed in the week of June 16. Prior to that week, the values are the number of farms testing positive for PEDV. Beginning with that week, the values are the number of diagnostic cases submissions, or accessions, and there may be many of these accessions for each infected farm. However, in the 9 weeks prior to June 16, the difference between numbers of positive case submissions and numbers of positive farms was typically less than 5. Thus it seems that the number of case submissions approximates the number of infected farms reasonably well. As mentioned in the main text, positive accessions are correlated with the number of positive farms available in more recent reports.

Predictors of cumulative burden

In the fitted regression models for cumulative burdens, many states in the Northeast had close centroids and very similar residuals, suggesting a lack of independence at this spatial scale. Using single linkage clustering, we found two groups of states that formed chains of states with centroids less than 175 km apart: (1) MD, DE, and NJ; and (2) VT, NH, CT, MA, and RI. Because of the lack of independence among data from these states suggested by our initial fits, we created a reduced data set where the values of both predictive variables and response variables for these two groups of states were averaged to form single observations. The results presented are based on that data, for which no spatial autocorrelation was indicated by maximum likelihood fits of a model of exponentially decaying covariance in the residuals.

Counts of farms of different sizes were obtained from a database application available from the USDA [5]. This application contains data from the 2007 Census of Agriculture.

The balance sheet variables for each state came from estimates for the period of December 2011 to December 2012 in Ref. [6]. These variables were swine inventory, which is the total number of swine; pig crop, which is the number of pigs born that survive the first few weeks of life; inshipments, which is the number of swine imported to the state; and marketings, which is the number of swine either exported from the state or slaughtered at a commercial facility.

Farm resource regions classify counties into one of 9 general groups based on a wide variety of criteria including farm characteristics and crops and livestock produced [7]. A list mapping counties to these regions was obtained from a USDA spreadsheet.[2] We included a variable for each region and each state was assigned a value in [0,1] for each such variable that was equal to the proportion of farms with 25 or more swine in the counties of that region.

Nearby positive accessions were calculated in various ways based on different possible models of spread. To represent cumulative exposure from a spatial model, we calculated for each state the average number of mean weekly positive accessions in other states with shared borders and used the logarithm of this average as a potential predictor. To represent cumulative exposure via shipment of pigs, we calculated for each state a weighted sum of mean weekly positive accessions in other states, where the weights were given by the flows from that state. To allow for nonlinear effects, we used various power transforms of the flows as weights. Specifically, we used the flows raised to the powers of 1, 1/2, 1/4, 1/8, and 1/16. These weighted sums were then log transformed to create a series of potential predictors.

The summary statistics for farm density were average number of farms per county, average number of farms in counties with at least one farm, median number of farms per county, and maximum number of farms per county. Some states had a median of zero farms per county. When log transforming, one half of

[2] `http://www.ers.usda.gov/Briefing/ARMS/resourceregions/reglink.xls`

the smallest positive median was added to values of all states before transformation. For this analysis, we defined farms as operations with 25 or more swine.

3. Simulations, correlation analysis, and sensitivity analysis

Simulations were run on a set of 1,000 points sampled from a Sobol sequence in the space of the continuous input variables. The one discrete input was the number of columns in the raster grid. Since generating the contact network was computationally intensive, we generated only one for each level of the variable and divided simulations evenly among them.

The spatial contact networks were generated as follows. We iterated over the count of swine farms by county in the 2002 census and randomly sampled coordinates for the location of the farm within the county. Over this set of points we overlaid a raster grid of square cells which covered the area of the contiguous United States. Farms with coordinates with the same cell or the Moore neighborhood of each other were connected with a spatial edge.

The transport contact networks were generated as follows. The goal was to generate an unweighted, directed network with a given mean degree and the total number of edges between farms in each pair of states proportional to the transport flows between those states. The number of edges between farms in the same state was to be proportional to the within state flows calculated for our time series regression model. We achieved this goal by dividing the elements of the transport flows matrix by the number of pairs of farms in the corresponding pairs of states and using the quotient as the probability of such an edge forming. These probabilities were scaled so that the expected number of edges was consistent with the target mean degree. Then a set of directed edges was sampled using a stochastic block model sampler which excluded edges that looped back to the farm of origin.

With the above contact networks generated and a set of parameters determined, time series of outbreaks by state were generated as follows. Our starting-grid input variables related to the location of the first outbreak of PED. The point of introduction of PEDV in 2013 is not known and so we were interested in seeing if this unknown variable had the potential to affect our results. The input variables determined which cell of a 10 cell wide by 2 cell tall grid over the contiguous United States the initial outbreak should occur in. For example, if the starting grid (x, y) coordinates were (0.05, 0.6), we sampled farms from the cell covering the state of Washington since 0.05 is in [0, 0.1) and 0.6 is in [0.5, 1). To begin the simulation, the farm selected by this procedure was classified as infected and all other farms were classified as susceptible. The status of all farms was determined at discrete steps corresponding to weeks according to the following algorithm. First, a seasonal adjustment factor was calculated as $1 - \{\text{seasonal amplitude}\} \sin(2\pi(x+3)/52)$ where x counts the number of steps completed plus one. Second the spatial, transport, and external transmission probabilities were all adjusted by multiplying the input values by the computed factor and resetting them to 1 if that caused them to exceed one. Third, susceptible farms were selected to be infected at the current step with a probability equal to the complement of no transmission occurring along any of edges leading to them and no transmission occurring to them from external sources. Finally, infected farms were selected to recover at the current step with a probability of 0.5. Thirty-seven updates were performed to generate a time series of length 38, which was the length of our empirical time series.

The outbreak time series was processed with an observation model based on our understanding of how the PED accession data were generated. From the simulations, we obtained a time series of newly infected farms in each weekly step. Since under-reporting may have been substantial for PED in 2013, we simulated the number of farms submitting samples to diagnostic labs each week for each state by sampling from a binomial distribution with a number of trials equal to the number of newly infected farms and with a probability of submission equal to 0.1. The number of accessions simulated was based on a negative binomial regression of the 2014–2015 accession counts on counts of presumptive and confirmed PEDV positive farms. Based on the estimates from that regression, we simulated the number of accessions by sampling from a negative binomial distribution with a mean of $0.75 + 1.92 \times \{\#\text{ farms submitting samples}\}$ and a dispersion parameter θ of 1.75. In this way, we generated a synthetic version of our empirical data set of weekly positive accessions by state.

Our synthetic and empirical data sets were used in a correlation analysis of the state-to-state similarities in the time series of positive accessions with the state-to-state proxy variables for contact rates. Similarity in time series was quantified by the the cross correlation with a lag of 1 week between all pairs of states with

any positive accessions. The cross correlation is the correlation between the values of one time series and corresponding values in another time series shifted by some lag. We conducted one-tailed Mantel tests with a significance threshold of $\alpha = 0.05$ to determine if there were significant positive correlations between corresponding elements of matrices of cross correlations, negative geographic distances, shared order indicator variables, and transport flows. The Mantel test evaluates the significance of such an association via a permutation procedure that accounts for the intrinsic dependence among elements of distance matrices[8]. This correlation analysis of the empirical data was followed up by two additional analyses described in the following subsections, but our simulation study was restricted to the correlation analysis to limit computational demands.

Since the simulated correlation for a given set of parameters is stochastic, we fit our simulation output to a metamodel to determine how the mean correlations changed with the inputs. We used a joint Gaussian process metamodel with the same covariance functions as Marrel and coauthors[9]. The parameters of each metamodel were optimized using an evolutionary algorithm[10] designed to avoid converging on local optima. Global sensitivity indices were calculated up to second order for all input parameters for the mean metamodel using standard Monte Carlo estimators [11]. We used a random Latin hypercube sample of 100,000 points from the metamodel to generate the estimates and calculated their confidence intervals using the basic bootstrap method. These global sensitivity indices quantified the sensitivity of the mean correlations to the input parameters without making any strong assumptions about the functional form of the relationship.

4. Age-specific reporting bias

Because infection mortality is high among piglets only [12], we might expect that operations without piglets are less likely to perform diagnostic testing. We can gain some insight into such potential reporting biases from the data about the age classes of diagnostic samples. These age classes are suckling (less than 1 month old or still on sow), nursery (1–3 months of age), grower/finisher (3–8 months), and sow/boar (more than 8 months old). Although the report providing the data uses the term age class and gives those particular age ranges for each class, these terms are really names for production stages in the swine industry for which there may be some variation outside of those ranges, in particular for the time at which pigs are weaned and sent to a nursery.

We tested the hypothesis that, among those states having any positive accessions with known age class, the age-class distribution is independent of the state of origin. We used simulation to generate a null distribution of test statistics rather than rely on asymptotic results because several of the observed cell counts were small. Tables of counts of samples in all combinations of age classes and states were simulated under the hypothesis of independence of age-class and state-of-origin. The simulated tables had the same marginal distributions as the observed data. The sum of Pearson residuals based on observed and expected cell counts was our test statistic. We conducted a test of independence at a significance level of 0.05. We rejected the hypothesis of independence based on an observed test statistic of 210, which was greater than the test statistic in all 10,000 of our null statistics ($p < 1 \times 10^{-4}$). To quantify the extent of dependence, we calculated that the uncertainty coefficient[13] of age class, given the state of origin, was equal to 0.05, which indicates relatively weak dependence.

To determine whether the proportion of positive accessions in the suckling age class may be explained by the distribution of farm types within a state, we compute expected proportions under a model of two-step random sampling as follows. In the first step, we sample a certain type of farming operation from a distribution of operation types. Table S1 gives the names of the available types. We obtain the distribution of these types for each state from census data[5]. In the second step we draw an age class from the age class distribution of the sampled farm type.

We derive an age-class distribution by first assuming that sows on average produced 2.31 litters of weaning size 10.3 every year and that pigs spent 21.5 days as suckling pigs, 46.0 days in the nursery stage, and 121.5 days in the grower/finisher stage. Those parameters are taken from 2012 averages from sow farms, nurseries, and conventional finishing farms participating in a U.S. benchmarking system [14, Tables 2, 4, and 5]. Larger farms tend to use artificial insemination [15, Table 3] and thus we assume that boars make up a negligible part of the total population on sow farms.

Given these parameters, we calculate an age-class distribution for the entire population by first calculating the rate of weaned pig production from the number of sows. The number of animals in all of the other age

classes follow as the product of that rate and the average time spent in each class. Age classes on a farms with some subset of age classes follow as a subset of the age-class distribution for the entire population to those classes present on the farm. Table S1 shows which age classes are typically present on each type of farm. We normalise these age-class distributions to obtain sampling probabilities conditional on a farm type.

We used a standard logistic regression analysis to test for an association between observed proportions of pigs in the suckling age class and those proportions predicted by our sampling model. The response variable was whether or not positive accessions where in the suckling age class and the predictors were probabilities from our sampling model and an intercept. We conducted a two-tailed Wald test of the hypothesis that the regression coefficient for the sampling probabilities was zero, and failed to reject this hypothesis ($p = 0.64$).

To see if the expected and observed probabilities were different on average, we fitted an intercept-only logistic model with logits of expected probabilities as offset terms. The observed log odds of suckling positive accessions was on average 3.54 natural logarithmic units above those predicted by random sampling (95% profile confidence interval = [3.15,3.97]). Removing highly influential observations (i.e., IA, NC, OK, KS, IL, and MN) resulted in somewhat lower interval estimate bounds of [1.86, 3.51]. These results indicate that farms with unweaned pigs are either more likely to choose unweaned pigs to be diagnostic samples than other pigs, more likely to seek laboratory confirmation of PEDV, or more likely to experience an outbreak than other farms.

5. Stability selection

We considered cumulative burdens to be an appropriate response variable because many of the candidate variables were not time-varying. Also, cumulative measures of burden may be more robust measures of incidence. Using the data on positive farms available after June 2014[16], we found the Spearman rank correlation between positive accessions and positive farms to equal 0.91, as compared to 0.74 for the weekly counts.

We used absolute burdens rather than prevalence as the response variable because of uncertainty in the correct denominator for calculation of prevalence. Our analysis of the positive accessions by age class in section 4 indicates that sampling of positive accessions may be highly biased toward farms with suckling pigs, which is reasonable because such farms would likely observe the most mortality in an outbreak[17]. However, we did not attempt to correct for this bias because we cannot rule out the possibility that in fact there was not bias but real increased risk to the farms with suckling pigs. Assuming that each time a trailer arrives for a pick-up there is a similar risk of infection, and that pigs typically spend about one month on sow-farms being weaned versus three months on finishing farms being fed to market weight, a sow farm of a certain size inventory would have a time-averaged risk 3-fold greater than a finishing farm of the same size inventory.

Many states had no confirmed positive accessions (Supplementary Fig. 1) such that the case counts appear to be a mixture of zeroes and a right-skewed distribution of counts. Thus we chose to fit the data to a hurdle model in which the probability of a state having a confirmed case and the number of positive accessions, given that there is at least one case in the state, are described by separate regression models. We used binomial generalised linear models for the probability responses and a least-squares linear model for the response of the log of positive accessions. Predictors were put onto the same scale by dividing by standard deviations.

The elastic net penalty includes a tuning parameter, denoted by α, that determines the extent to which groups of correlated variables are selected together. We set α to 0.8 to allow for highly correlated variables to be grouped for selection while still keeping the total number of selected variables small.

The choice $\alpha = 0.8$ was made subjectively, but we checked that the results were not sensitive to this choice by also looking at the results with $\alpha \in \{0.01, 0.2, 0.5, 1\}$. For $\alpha \neq 1$, only additional balance sheet variables were selected for all models. When $\alpha = 1$, inventory and resource region 4 were selected as predictors of both litter rate decrease and total positive accessions, and no variables were selected as predictors of whether any positive accessions occurred. We consider these aberrations likely to be an artefact of correlations among predictors, as single members of correlated groups can be selected somewhat arbitrarily when $\alpha = 1$.

For stability selection, we used 1,000 subsamples of 63.2 percent of the full data sets (the same percentage that would appear in large bootstrap samples of a data set). The set of selected variables was chosen by using a threshold parameter π_{thr} of 0.6 and choosing the regularisation parameter λ to select as many variables as

possible while keeping the per-comparison error rate (i.e., the probability that any one variable is incorrectly selected) below 0.05. The results of stability selection are not usually sensitive to the choice of π_{thr} as long as it is between 0.6 and 0.9. The error rate is only guaranteed to hold under the restrictive assumption of exchangeability for the selection probability of all noise variables, but numerically it has been found to be accurate even when this assumption was most likely not satisfied [18]. Although we cannot guarantee similar accuracy for our data set, we propose that controlling the nominal error rate provides a reasonable criteria for identifying the candidate variables that are most likely to be relevant.

6. Time series regression modelling

A transmission model is integrated within a regression model by having the expected number of outbreaks in state i at week $t+1$, $\mathrm{E}(I_{i,t+1})$ follow

$$\mathrm{E}(I_{i,t+1}) = \beta_{i,t}(\textstyle\sum_j w_{i,j} I_{j,t} + \eta)^{\alpha} S_{i,t}, \tag{S1}$$

where $\beta_{i,t}$ is the transmission rate for state i at time t, $w_{i,j}$ is the weight for the influence of infectives in state j on susceptibles in state i, η is parameter that determines the influence of other sources of infection, α determines the power by which the expected number of transmissions grows with these risks, and $S_{i,t}$ is the number of susceptibles in state i at week t. We set $S_{i,t} = N_i - \sum_{k=0}^{t-1} I_{i,t}$, where N_i is the number of farms in state i from the 2002 Census of Agriculture[19]. This model is a variant of the time series SIR (susceptible–infective–recovered) model[20].

A number of simplifying assumptions are implicit in equation S1. First, we treat entire farms as either infective or susceptible. Second, we assume that the infection of a farm lags 1 week behind its infectious exposure. In support of this assumption, we found that a 1-week lag had a higher likelihood in our models than lags of 2 to 4 weeks. Third, we assume that farms are only infectious for 1 week. This assumption is a simplification that may not be too inaccurate if farms are most infectious the first week of an outbreak, perhaps because the number of animals shedding later becomes smaller or because more stringent biosecurity reduces the amount of infectious material leaving the farm. This assumption is congruent with those made by Ref. [21, p. 71] in setting parameters for an agent-based model of spread.

For the number of farms N_i, we used data from the 2002 Census [19] instead of data from more recent censuses so as to obtain farm count data that were contemporary with the transport flow data, which are from 2001 [2]. In this analysis, we included farms with any swine in the counts, unlike our analysis of cumulative burdens where we only included farms with at least 25 swine. All farms were included here because farms with fewer than 25 swine are numerous enough to constitute a non-negligible fraction of total swine inventory and flows.

Our calculation of $S_{i,t}$ assumes that all farms were susceptible to infection at the beginning of the epizootic and that farms pass on to an immune state following infection. The assumption of complete susceptibility seems reasonable for the United States given the absence of previous reports of PED and the high frequency of high-mortality outbreaks that followed the first reported outbreak[22]. Although PED has been observed to reoccur on a farm[23], that observation was a newsworthy event[24] and it followed a 6-month interval of normal operations. Thus the assumption of immunity over the 38 week period that we analyse seems reasonable.

Our transmission rate $\beta_{i,t}$ in equation (S1) takes the form

$$\beta_{i,t} = \exp(c_0 + Z_i + c_1 t)(N_i^2 d_i)^{c_2} f_i^{c_3} N_i^{-2}, \tag{S2}$$

where the c_i are unknown parameters that we estimate, Z_i represents state-level random effects, d_i is a state-level summary statistic of the county-level farm density from the 2007 Census [5], and f_i is value characterising the average flow of swine through individual farms in state i. c_1 allows the transmission rate to vary seasonally, which has been proposed as an explanation for why most positive accessions occurred in the fall and winter. For the summary statistic d_i, we used the median county-level density among counties with any farms in the state. The results were not sensitive to using this statistic versus others such as the overall median or mean. d_i is multiplied by N_i^2 because that led to the greatest correlation between the density and flow terms on the logarithmic scale, and we wished to as much as possible separate the estimated effects of flows with those of farm density. It also allowed us to see whether density-dependent transmission [25] is suggested by the data, which would have corresponded to estimates $(\hat{c}_2, \hat{c}_3) \approx (1, 0)$.

The characteristic flows f_i in equation (S2) and the weights w_i in equation (S1) are calculated in various ways to model the rate of contact of a susceptible farm with infected farms in various scenarios. We make the derivations assuming $\alpha = 1$, and values of α below 1 can be understood as capturing the effects of infective farms being clustered together in the contact network. Let $F_{i,j}$ be the number of swine shipped to farms in state i from farms in state j per year. In the *directed model,* only farms receiving animals are at risk for infection. Then, omitting the time subscripts for simplicity, susceptible farms in state i are infected at a rate proportional to $\sum_j F_{i,j}(N_i N_j)^{-1} I_j$, or $f_i N_i^{-2} \sum_j w_{i,j} I_j$, where $f_i = \sum_j F_{i,j}$ and $w_{i,j} = N_i N_j^{-1} F_{i,j} f_i^{-1}$. In the *undirected model,* both farms sending and farms receiving animals may be at risk, and susceptible farms in state i are infected at a rate proportional to $\sum_j (F_{i,j} + F_{j,i})(N_i N_j)^{-1} I_j$, which implies that $f_i = \sum_j F_{i,j} + F_{j,i}$ and $w_{i,j} = N_i N_j^{-1}(F_{i,j} + F_{j,i}) f_i^{-1}$.

In the *internal model,* both farms sending and receiving animals may be at risk, but transmission associated with flows only occurs within a state. Susceptible farms in state i are infected at a rate proportional to $2F_{i,i} N_i^{-2} I_i$. In this case, $f_i = 2F_{i,i}$ and $w_{i,j} = \delta_{i,j}$, where $\delta_{i,j}$ is a Kronecker delta. Comparison of the fit of this model with the directed or undirected models allows any effects of between-state transmission to be seen. The internal model also includes in the case that $c_3 = 0$ a null model which has no flows in it, which we use in a likelihood ratio test of the hypothesis that flows have no effect on transmission rates.

The values of $F_{i,j}$, when $i \neq j$, come directly from the estimates[2] of interstate flows. We estimated within-state flows in two ways. In the first, a demand for pigs was calculated for state i from 2002 sales[19] of finish-only and nursery operations plus the deaths reported in the 2001 balance sheet[26]. Internal flow, $F_{i,i}$, was estimated as the this demand less imports, $\sum_{j, j \neq i} F_{i,j}$. In the second method, $F_{i,i}$ was estimated as the combined sales of farrow-to-wean, farrow-to-feeder, and nursery operations less exports, $\sum_{j, i \neq j} F_{j,i}$. For most states with large inventories, the logarithms of these two estimates were similar relative to estimates from other states, and we averaged the log-transformed estimates to generate a single estimate. For the other states, one of the estimates was negative, and we simply used the positive estimate. We suspect the negative estimates and the difference between the positive estimates stem in part from us not being able to use 2001 sales data or to account for internal supplies of and demand for breeding animals. Coarse as these estimates may be, it still seems reasonable to us that they will permit detection of large, state-level effects on transmission rates. To that end, we formed linear predictor of $\log \mathrm{E}(I_{i,t+1})$ by substituting equation (S2) into equation (S1) and taking logarithms to obtain equation 1 in the Methods of the main text and proceeded as described there.

7. Software

We used R[3] for most of this work. The key contributed packages used were c060[27], DiceKriging[28], igraph[29], glmmADMB[30], glmnet[31], ggplot2[32], lme4[33], rgenoud[10], sensitivity[34], sp[35], and vegan[36]. We performed the edge bundling for Supplementary Figs. 1 using JFlowMap [37]. Code to reproduce the results is archived on the web[38], and has been developed to run in Docker[39] containers for enhanced reproducibility. Thus, after installing one open-source software package on their personal computer, interested readers may quickly repeat our analysis, examine intermediate results, perform their own diagnostics, and extend this work.

References

[1] Höhle, M., Meyer, S. & Paul, M. *surveillance: Temporal and Spatio-Temporal Modeling and Monitoring of Epidemic Phenomena* (2015). R package version 1.10-0. `http://CRAN.R-project.org/package=surveillance`.

[2] USDA ERS. Interstate livestock movements. By D. Shields and K. Mathews. Available: `http://www.ers.usda.gov/publications/ldpm-livestock,-dairy,-and-poultry-outlook/lpdm10801.aspx#.U26fN1Qt5Mk` (2003). Accessed 14 November 2013.

[3] R Core Team. *R: A Language and Environment for Statistical Computing.* R Foundation for Statistical Computing, Vienna, Austria (2014).

[4] USDA APHIS VS. Porcine epidemic diarrhea virus (PEDv) testing data from NAHLN laboratories (2014). Available: http://www.aasv.org/pedv/PEDV_weekly_report_140108.pdf. Accessed 14 January 2014.

[5] USDA NASS. 2007 Desktop Data Query Tool 1.02 (2009). Available: http://www.agcensus.usda.gov/Publications/2007/Online_Highlights/Desktop_Application/dataquery.zip. Accessed 14 January 2013.

[6] USDA NASS. Meat Animals Production, Disposition, and Income 2012 Summary (2013). Available: http://usda01.library.cornell.edu/usda/current/MeatAnimPr/MeatAnimPr-04-25-2013.zip. Accessed 31 July 2013.

[7] USDA ERS. Farm Resource Regions. Available: http://www.ers.usda.gov/ersDownloadHandler.ashx?file=/media/926929/aib-760_002.pdf (2000). Accessed 3 April 2014.

[8] Sokal, R. R. & Rohlf, F. J. *Biometry* (W. H. Freeman and Company, 2001), 3 edn.

[9] Marrel, A., Iooss, B., Veiga, S. D. & Ribatet, M. Global sensitivity analysis of stochastic computer models with joint metamodels. *Stat Comput* **22**, 833–847 (2011).

[10] Sekhon, J. S. & Mebane, Jr., W. R. Genetic optimization using derivatives: Theory and application to nonlinear models. *Polit Anal* **7**, 189–213 (1998).

[11] Sobol', I. M. Global sensitivity indices for nonlinear mathematical models and their Monte Carlo estimates. *Math Comput Simul* **55**, 271–280 (2001).

[12] USDA. Summary of PEDV Actions. Available: http://www.usda.gov/documents/pedv-summary-actions.pdf (2014). Accessed 10 May 2014.

[13] Tang, W., He, H. & Tu, X. M. *Applied Categorical and Count Data Analysis* (CRC Press, 2012).

[14] Kenneth J. Stalder. Pork industry productivity analysis. Available: http://old.pork.org/filelibrary/research/ipafull.pdf (2013). Accessed 2 December 2014.

[15] USDA ERS. The changing economics of U.S. hog production. By N. Key and W. McBride (2007). ERR-52. Available: http://www.ers.usda.gov/media/244843/err52.pdf. Accessed 2 December 2014.

[16] USDA APHIS VS. Swine enteric coronavirus disease (SECD) situation report Feb 12, 2015 (2015). Available: http://www.aphis.usda.gov/animal_health/animal_dis_spec/swine/downloads/secd_sit_rep_02_12_15.pdf. Accessed 14 Februrary 2014.

[17] Jung, K. & Saif, L. J. Porcine epidemic diarrhea virus infection: etiology, epidemiology, pathogenesis and immunoprophylaxis. *Vet J* **204**, 134–143 (2015).

[18] Meinshausen, N. & Bühlmann, P. Stability selection. *J R Stat Soc Series B Stat Methodol* **72**, 417–473 (2010).

[19] USDA NASS. 2002 Census of Agriculture Query Tool. Available: http://www.agcensus.usda.gov/Publications/2002/Download_Data_Query_Application/agcensus2002.zip. Accessed 23 December 2014.

[20] Bjørnstad, O. N., Finkenstädt, B. F. & Grenfell, B. T. Dynamics of measles epidemics: Estimating scaling of transmission rates using a time series SIR model. *Ecol Monogr* **72**, 169–184 (2002).

[21] ANSES. Relatif au risque d'émergence de la diarrhée épidémique porcine (DEP) due à un nouveau variant du virus de la DEP en France. Available: https://www.anses.fr/fr/system/files/SANT2014sa0087.pdf. Accessed 14 December 2015.

[22] EFSA AHAW Panel. Scientific opinion on porcine epidemic diarrhoea and emerging pig deltacoronavirus. *EFSA Journal* **12**, 3877 (2014).

[23] Ackerman, M. A. PEDv recurrence. Available: https://www.pig333.com/clinical-case-of-the-world/pedv-recurrence_9260/ (2014). Accessed 25 January 2015.

[24] Polansek, T. Exclusive: Deadly pig virus re-infects U.S. farm, fuels supply fears. Available: http://www.reuters.com/article/2014/05/28/us-pig-virus-immunity-idUSKBN0E811N20140528 (2014). Accessed 10 December 2014.

[25] Begon, M. *et al.* A clarification of transmission terms in host-microparasite models: numbers, densities and areas. *Epidemiol Infect* **129**, 147–153 (2002).

[26] USDA NASS. Meat Animals Production, Disposition, and Income 2001 Summary (2002). Available: http://usda.mannlib.cornell.edu/usda/nass/MeatAnimPr//2000s/2002/MeatAnimPr-04-26-2002.zip. Accessed 23 December 2014.

[27] Sill, M., Hielscher, T., Becker, N. & Zucknick, M. c060: Extended inference with lasso and elastic-net regularized Cox and generalized linear models. *J Stat Softw* **62**, 1–22 (2014).

[28] Roustant, O., Ginsbourger, D. & Deville, Y. DiceKriging, DiceOptim: Two R packages for the analysis of computer experiments by kriging-based metamodeling and optimization. *J Stat Soft* **51**, 1–55 (2012).

[29] Csardi, G. & Nepusz, T. The igraph software package for complex network research. *InterJournal* **Complex Systems**, 1695 (2006).

[30] Skaug, H., Fournier, D., Bolker, B., Magnusson, A. & Nielsen, A. *Generalized Linear Mixed Models using AD Model Builder* (2014).

[31] Friedman, J., Hastie, T. & Tibshirani, R. Regularization paths for generalized linear models via coordinate descent. *J Stat Softw* **33**, 1–22 (2010).

[32] Wickham, H. *ggplot2: Elegant Graphics for Data Analysis* (Springer New York, 2009).

[33] Bates, D., Maechler, M., Bolker, B. & Walker, S. *lme4: Linear mixed-effects models using Eigen and S4* (2014).

[34] Pujol, G., Iooss, B., Janon, A. & contributers. *sensitivity: Sensitivity Analysis* (2015).

[35] Pebesma, E. J. & Bivand, R. S. Classes and methods for spatial data in R. *R News* **5**, 9–13 (2005).

[36] Oksanen, J. *et al. vegan: Community Ecology Package* (2015).

[37] Boyandin, I., Bertini, E. & Lalanne, D. Using flow maps to explore migrations over time. In *Proceedings of Geospatial Visual Analytics Workshop in conjunction with The 13th AGILE International Conference on Geographic Information Science (GeoVA)* (Guimaraes (Portugal), 2010).

[38] O'Dea, E. 2015pedv: Files associated with Dec. 20 draft. Available: http://dx.doi.org/10.5281/zenodo.35573 (2015).

[39] Boettiger, C. An introduction to Docker for reproducible research. *SIGOPS Oper. Syst. Rev.* **49**, 71–79 (2015).
